# Clinical and laboratorial impact of antiretroviral therapy in a cohort of Portuguese patients chronically infected with HIV-2

**DOI:** 10.7448/IAS.17.4.19829

**Published:** 2014-11-02

**Authors:** Ana Miranda, Susana Peres, Virginia Moneti, Telma Azevedo, Isabel Aldir, Kamal Mansinho

**Affiliations:** Serviço de Infecciologia e Medicina Tropical, Hospital Egas Moniz, Centro Hospitalar de Lisboa Ocidental, Lisbon, Portugal

## Abstract

**Introduction:**

HIV-2 infection is endemic in West Africa and some European countries, namely Portugal. HIV-2 antiretroviral (ARV) treatment presents some restrains related to intrinsic resistance to non-nucleoside reverse transcriptase inhibitors (NNRTI) and fusion inhibitors, and poorer response to protease inhibitors (PI).

**Material and Methods:**

Retrospective observational study of a cohort of 135 infected HIV-2 patients, diagnosed between 1989 and 2008.

**Objectives:**

Evaluation of epidemiologic, clinical, immunologic and virologic progression, comparing to groups of patients (naïve vs ARV experienced); characterization of therapeutic, immunologic and virologic response. SPSS version 20.0 was used for statistical analysis.

**Results:**

The study included 135 patients: 41% (n=55) naïve and 59% (n=80) with ARV experience. The comparison between groups (naïve vs ARV) revealed: male prevalence 76% vs 50%; mean age 54.5 years vs 54.8 (p=0.90); main geographic origin Guiné Bissau (47% vs 44%) and Portugal (22% vs 33%); and transmission mainly acquired by heterosexual contact (87% vs 80%). Mean time since diagnosis was 14 vs 13 years (p=0.31); 2% vs 50% presented AIDS criteria at diagnosis (p<0.001) and 93% vs 38% registered TCD4>350 cell/mm^3^ at diagnosis (p<0.001). Immunological evolution showed no significant decline in naïve population (Δ=−67 cell/mm^3^ – p=0.18) and a significant recovery in ARV experienced (Δ=+207 cell/mm^3^ – p<0.001). Global mortality rate found was 18% (6% vs 13% – p=0.122). Eighty patients initiated ARV: 84% presented a time interval of ARV exposure between 0–5 years (42%) and 5–10 years (42%). Fifty percent experienced ≤2 ARV regimens and the remaining >2 regimes. Considering the first ARV therapy: 56% initiated PI, 30% NTRI and 5% integrase inhibitor (II)-based regimens. Currently, 54 patients maintain regular follow-up and ARV therapy: 60% NTRI+PI; 37% NRTI+PI+II and 3% NRTI+II. TDF/FTC is the backbone in 56%. Most frequent PIs are LPV/r (54%), DRV/r (19%) and ATV/r (12%). Mean time of exposure to NRTI=3 years, PI=7 years and II=2 years. Immunologic recovery was sustained for each of the ARV class considered (NRTI Δ=+144 cell/mm^3^; PI=Δ+92 cell/mm^3^; II=Δ=+116 cell/mm^3^).

**Conclusions:**

This is a cohort accompanied for a long period and the majority of patients present extensive ARV experience. The ARV-experienced patients registered a favourable response to treatment, with sustained immune recovery (Δ=+207 cell/mm^3^) and virologic control in 74%. Immunologic behaviour evidenced a sustained gain for each of the ARV class considered.

